# Hyaluronic acid in tooth extraction: a systematic review and meta-analysis of preclinical and clinical trials

**DOI:** 10.1007/s00784-023-05227-4

**Published:** 2023-11-15

**Authors:** Danijel Domic, Kristina Bertl, Tobias Lang, Nikolaos Pandis, Christian Ulm, Andreas Stavropoulos

**Affiliations:** 1 Division of Oral Surgery, University Clinic of Dentistry, Medical University Vienna, Sensengasse 2a, 1090 Vienna, Austria; 2 Department of Periodontology, Dental Clinic, Faculty of Medicine, Sigmund Freud University, Freudplatz 3, 1020 Vienna, Austria; 3 Periodontology, Faculty of Odontology, University of Malmö, Carl Gustafs Väg 34, 205 06 Malmö, Sweden; 4 Department of Orthodontics and Dentofacial Orthopedics, School of Dental Medicine, University of Bern, Freiburgstrasse 7, 3010 Bern, Switzerland; 5 Division of Conservative Dentistry and Periodontology, University Clinic of Dentistry, Medical University of Vienna, Sensengasse 2a, 1090 Vienna, Austria; 6 Department of Periodontology, School of Dental Medicine, University of Bern, Freiburgstrasse 7, 3010 Bern, Switzerland

**Keywords:** Hyaluronic acid, Tooth extraction, Wound healing, Systematic review

## Abstract

**Objectives:**

To assess whether in animals or patients with ≥ 1 tooth extracted, hyaluronic acid (HyA) application results in superior healing and/or improved complication management compared to any other treatment or no treatment.

**Materials and methods:**

Three databases were searched until April 2022. The most relevant eligibility criteria were (1) local application of HyA as adjunct to tooth extraction or as treatment of alveolar osteitis, and (2) reporting of clinical, radiographic, histological, or patient-reported data. New bone formation and/or quality were considered main outcome parameters in preclinical studies, while pain, swelling, and trismus were defined as main outcome parameters in clinical studies.

**Results:**

Five preclinical and 22 clinical studies (1062 patients at final evaluation) were included. In preclinical trials, HyA was applied into the extraction socket. Although a positive effect of HyA was seen in all individual studies on bone formation, this effect was not confirmed by meta-analysis. In clinical studies, HyA was applied into the extraction socket or used as spray or mouthwash. HyA application after non-surgical extraction of normally erupted teeth may have a positive effect on soft tissue healing. Based on meta-analyses, HyA application after surgical removal of lower third molars (LM3) resulted in significant reduction in pain perception 7 days postoperatively compared to either no additional wound manipulation or the application of a placebo/carrier. Early post-operative pain, trismus, and extent of swelling were unaffected.

**Conclusions:**

HyA application may have a positive effect in pain reduction after LM3 removal, but not after extraction of normally erupted teeth.

**Clinical relevance:**

HyA application may have a positive effect in pain reduction after surgical LM3 removal, but it does not seem to have any impact on other complications or after extraction of normally erupted teeth. Furthermore, it seems not to reduce post-extraction alveolar ridge modeling, even though preclinical studies show enhanced bone formation.

**Supplementary Information:**

The online version contains supplementary material available at 10.1007/s00784-023-05227-4.

## Introduction

Although the healing process following tooth extraction is commonly uneventful, any subsequent pain may compromise patients’ well-being, while complications may also occur. For example, surgical extraction of semi-/fully impacted third molars is regularly associated with significant pain, swelling, and trismus [1–3], which are aggravated in case of development of alveolar osteitis (AO)—also called dry socket. AO is considered one of the most frequent complications of tooth extraction occurring in 20 to 35% of the cases of surgical extraction of lower third molars (LM3), and in 1.4 to 5% of (non-surgical) extraction of regularly erupted teeth [1, 2, 4]. Besides such early complications, which negatively affect patients’ quality of life, compromised extraction socket healing may also lead to significant hard tissue defects, either at the extraction site or at the neighboring teeth [5, 6]. For example, it has been reported that deep periodontal defects, e.g., probing pocket depths ≥ 7 mm, at the distal aspect of the second molar occur in almost every fourth patient after extraction of impacted LM3 [5].

To reduce patient morbidity and improve soft and hard tissue healing of extraction sockets, as well as for the treatment of early complications (e.g., AO), various materials and/or surgical techniques have been tested (e.g., application of collagen sponges, gels, blood derivates, various grafting materials) [7–9]. Increasing attention is recently put on hyaluronic acid (HyA), due to its anti-inflammatory and antibacterial properties [10–12] and its positive effects on soft and hard tissue healing. Specifically, preclinical studies have demonstrated a positive effect, histologically, on the healing of bone [13, 14] and periodontal defects [15, 16] after HyA application. Based on the results of the meta-analyses of a systematic review of clinical trials on surgical extraction of third molars, significantly reduced pain on the third and seventh postoperative day, but not on trismus, was reported in groups receiving HyA-based products [17]. In this context, a comprehensive assessment of the available preclinical and clinical evidence on the effect of HyA application in connection with tooth extraction in general, including the prevalence, extent, and/or management of complications is missing. Therefore, the present systematic review addressed the following PICOS (population (P), intervention (I), comparison (C), outcomes (O), and study design (S)) question: “In animals/patients having ≥ 1 tooth extracted, does application of HyA alone or combined with other products/carriers result in superior soft-/hard tissue healing, reduced morbidity, reduced complication rate, and/or improved complication management compared to any other treatment or no treatment?”.

## Material and methods

### Study protocol and study registration

The present work followed available guidelines for performing systematic reviews of preclinical [18] and clinical studies (Preferred Reporting Items for Systematic Reviews and Meta-analysis (PRISMA); Appendix 1) [19]. Both protocols were registered at the international prospective register of systematic reviews (PROSPERO), i.e., one for the preclinical (CRD42021266190) and one for the clinical trials (CRD42021266183).

### Information sources, literature search, and eligibility criteria

The literature search was performed in 3 databases (i.e., Ovid (MEDLINE and CENTRAL), EMBASE, and Pubmed)) on October 14, 2021, and updated on April 7, 2022. Details on the search including the keywords are presented in Appendix 2. After removing the duplicates, titles and abstracts were screened for eligibility by 2 reviewers (DD, TL) and kappa values for the screened full texts and finally included publications were calculated. Any ambiguity was resolved in discussion with a third author (KB). Independent of the study type, studies were included if (a) written in English or German language, (b) the full text was available, and (c) clinical, radiographic, or histological data were provided. Additional inclusion criteria for the preclinical studies were (a) randomized and non-randomized controlled experiments, and (b) local application of a HyA-based product alone or in combination with another product in ≥ 1 of the groups after extraction of ≥ 1 tooth. Additional inclusion criteria for the clinical studies were (a) randomized controlled trial (RCT), controlled trial (CT), or case series with a minimum of 10 patients, and (b) local application of a HyA-based product alone or in combination with another product in ≥ 1 of the groups either after extraction of ≥ 1 tooth or as treatment of AO of ≥ 1 tooth.

### Data collection and extraction

Two authors (DD, KB) independently extracted the data twice and any disagreement was resolved in discussion with a third author (AS). From the preclinical studies, the following information was extracted: (a) first author, (b) publication year, (c) study design, (d) treatment model, (e) treatment site, (f) species, (g) HyA application form, (h) treatment groups, (i) follow-up period, (j) available outcome parameters, and (k) funding details. Similarly, the following information was extracted from the clinical trials: (a) first author, (b) publication year, (c) study design, (d) patient characteristics (i.e., gender, age, health and smoking status), (e) site-specific inclusion criteria, (f) number of sites at baseline and last follow-up, (g) treatment groups, (h) product details, (i) application form, (j) follow-up period, (k) postoperative medication, (l) available outcome parameters, (m) clinical setting (i.e., private practice or university setting), and (n) funding details. Finally, all available information on the HyA-based products was summarized, i.e., (a) trade name, (b) manufacturer, (c) concentration, (d) chemical form, and (e) application form.

### Risk of bias assessment

For the preclinical trials, the SYRCLE’s risk of bias (RoB) tool was used [20]. As suggested, the following criteria were evaluated as having “low,” “high,” or “unclear” RoB: (1) sequence generation, (2) baseline characteristics, (3) allocation concealment, (4) random housing, (5) blinding caregivers or researchers, (6) random outcome assessment, (7) blinding outcome assessor, (8) incomplete outcome data, (9) selective outcome reporting, and (10) other sources of bias. For each study, the number and percentage of positively scored items were calculated (i.e., “quality score”).

For the RCT, the Cochrane Collaboration’s RoB 2.0 tool was used [21]. The RoB was judged as having “low,” “high,” or “some” concerns for each of the following criteria: (1) randomization process, (2) deviations from intended interventions, (3) missing outcome data, (4) measurement of the outcome, (5) selection of the reported result, and (6) overall risk of bias. For the non-randomized trials, the ROBINS-I tool was used [22]. The risk of bias was judged as “low,” “moderate,” “serious,” “critical,” or “no information” for the following criteria: (1) confounding, (2) selection of participants, (3) classification of interventions, (4) deviations from intended interventions, (5) missing outcome data, (6) measurement of the outcome, (7) selection of the reported result, and (8) overall risk of bias.

The assessment was done by 2 reviewers (DD, KB), and in case of any ambiguity consensus was achieved by discussion with a third author (AS). One author repeated the assessment (DD).

### Synthesis of results and statistical analysis

For the preclinical studies, new bone formation and bone volume per tissue volume (BV/TV) were considered main outcome parameters, while for the clinical studies pain, trismus, and swelling were defined as main outcome parameters. Data were extracted from the text, tables, and figures, calculated, and/or the authors of the original publications were contacted.

In case at least 2 randomized studies with comparable study design (i.e., treatment indication, HyA regime, follow-up period, outcome assessment) were identified, a pairwise meta-analysis was performed. The meta-analyses were limited to RCTs, thus including studies of greater methodological quality. The groups applying HyA were either compared to a negative control group (i.e., with no additional treatment step) or to a control group applying another treatment, including a placebo or the carrier material of the test group (“placebo/carrier”). Pairwise meta-analyses were performed for each separate comparison as well as overall. Restricted maximum likelihood to calculate heterogeneity (τ2) was used and the Knapp–Hartung standard error adjustment to account for the small number of studies. The mean difference between control and test group, the standard error of the mean difference, and 95% confidence interval (CI) were calculated. In studies using split-mouth design, the data were treated as dependent when calculating the standard error of the mean difference with setting *r* = 0.5. The chi-square test was used to assess heterogeneity, and a *p*-value < 0.1 was considered indicative of significant heterogeneity [23]. Further, *I*^2^ test for homogeneity was undertaken to quantify the extent of heterogeneity and in case of at least 3 comparable studies the 95% prediction interval was additionally calculated. Statistical analysis was performed with STATA/IC 17.0 for Mac.

### *Quality of evidence (GRADE)*

The certainty of meta-analytic evidence of preclinical and clinical trials included herein was summarized by Grading of Recommendations Assessment, Development and Evaluation (GRADE) [24, 25]. For both preclinical and clinical trials, the GRADEpro GDT (Guideline Development Tool, McMaster University and Evidence Prime, 2022) software was used to grade the quality of evidence of the results.

## Results

### Study selection and characteristics

The literature search is presented in the Appendix 3; 147 potential references were identified and, after removing the duplicates, 90 studies were left for title and abstract screening. A total of 57 studies were removed for various reasons leaving 33 studies for full text analysis. After excluding another 6 studies in which the product type did not meet the inclusion criteria or incorrect study design [26–29], 5 preclinical and 22 clinical studies were included in the present systematic review. Both reviewers agreed perfectly on studies chosen for full-text screening (Cohen’s kappa = 1; 100% agreement), while substantial agreement was achieved for final study enrollment (Cohen’s kappa = 0.61; 84.9% agreement).

In all preclinical trials, HyA was applied into the tooth socket after extraction of regularly erupted teeth [30–34]. The clinical trials were divided into 3 groups according to treatment indication: (1) surgical removal of LM3 (RCT (*n* = 10), CT (*n* = 1)) [35–45], (2) extraction of regularly erupted teeth (RCT (*n* = 7), non-randomized split-mouth study (*n* = 1), prospective case series (*n* = 1)) [46–54], and (3) treatment of AO (RCT (*n* = 1), prospective case series (*n* = 1)) [55, 56].

### Study population

Regarding the preclinical studies, 2 studies included 5–11 Holtzman or 5–6 Wistar rats in the various groups, respectively [30, 31], while 3 studies used beagle dogs (20 dogs in total) [32–34]. In the rat studies, HyA was applied in the extraction socket in either healthy or diabetic animals, whereas in the dog studies HyA was applied in infected extraction sockets (Table 1).

**Table 1:** Details of the included preclinical studies

Study (year) Study design	Treatment model Treatment site	Species	Application form	Treatment groups	Follow-up	Outcome parameters
Mendes (2008) Randomized	Extraction socket UM1	Holtzman rats	1% HyA gel (Nikkol; intraoperative)	HyA	1st–5th, 7th, 21st day	BMP-2 and OPN expression Bone formation
				Carbopol		
				Blood clot		
Sa (2013) Non-randomized	Extraction socket in healthy and diabetic specimen UM1	Wistar rats	0.25, 0.5, 1, 2, 4% HyA gel (Galena; intraoperative)	Carbopol (non-diabetic)	7th, 14th day	Bone formation
				Carbopol (diabetic)		
				HyA (diabetic)		
				HyA + SWCNT (diabetic)		
Kim (2016) Randomized	Infected extraction socket LM3	Beagle dogs	1% HyA gel (Healon; intraoperative)	HyA	3rd month	Bone formation
				Blood clot		
Kim (2019) Randomized	Infected extraction socket LPM3, LPM4	Beagle dogs	1% HyA gel (Healon; intraoperative)	ACS	3rd month	Bone formation
				HyA + ACS		
				rhBMP-2 + ACS		
				Blood clot		
Lee (2021) Randomized	Infected extraction socket LPM3, LPM4, LM1	Beagle dogs	1% HyA gel (Healon; intraoperative)	ACS	1st, 3rd month	Bone formation
				HyA + ACS		
				DBBM-C		
				HyA + DBBM-C		

*ACS* absorbable collagen sponge, *BMP* bone morphogenetic protein, *DBBM-C* deproteinized bovine bone mineral with collagen, *HyA* hyaluronic acid, *LM3* lower third molar, *LPM3/4* lower third/fourth premolar, *OPN* osteopontin, *rhBMP* recombinant human bone morphogenetic protein, *SWCNT* single-walled carbon nanotube, *UM1* upper first molar

The clinical studies on surgical LM3 removal, extraction of regularly erupted teeth, and treatment of AO included at final evaluation 603, 349, and 110 patients, respectively, contributing with 306, 226, and 90 HyA treated sites, and 370, 257, and 20 control/non-HyA treated sites, respectively (Table 2). In most of the studies, patients were systemically healthy, while one study each regarded patients with either chronic liver disease or diabetics; 4 studies did not report on patients’ health status. Smoking status was reported in 12 studies; 8 studies included only non-smokers, 2 studies included patients smoking ≤ 10 cigarettes/day, and 2 studies included both, i.e., non-smokers and smokers. Ten studies did not provide any information on smoking status.

**Table 2:** Details of the included clinical studies

Study (year) Study design	Patient characteristics Gender (f/m) Age (years) Health status Smoking status	Inclusion criteria (site)	Site number (no. at baseline/follow-up)	Treatment groups and product details	Application form	Follow-up	Postoperative medication	Outcome parameters
			Test	Test				
			Control	Control				
Lower third molar (LM3)								
Koray (2014) RCT, split-mouth	15/19 23.4 ± 3.9 Healthy NR	Bilateral, symmetrically impacted LM3 with total or partial bone cover and comparable surgical difficulty	34/34	50 ml Gengigel HyA spray (0.2%)	Spray 3 × /day for 7 days	2nd, 7th day	1 g amoxicillin 2 × 1; 550 mg naproxen 3 × 1 for 4 days	Edema, pain (VAS), trismus
			34/34	BnzHCl spray				
Gocmen (2015) RCT	20/20 26.6 ± 6.3 ASA I-II NR	Vertically positioned, erupted/half impacted LM3 without bone retention	20/20	0.2 ml Gengigel Prof HyA gel (0.8%)	Intra-operative	7th day	NR	Pain (VAS), trismus, inflammatory response, oxidative stress
			20/20	Nothing				
Gocmen (2017) RCT	NR 24.8 ASA I-II Non-smoker	Vertical, half impacted LM3 without bone retention	20/20	0.2 ml Gengigel Prof HyA gel (0.8%)	Intra-operative	1 h, 3rd, 7th day	1 g amoxicillin 2 × 1 for 7 days; ibuprofen 400 mg 4 × 1 for 2 days	Trismus, bleeding time, tissue factor, edema, pain (VAS)
			20/20	Nothing				
Yilmaz (2017) CT, split-mouth	12/13 21.2 ± 3.0, > 18 Healthy Non-smokers	Bilaterally impacted LM3 (class 3-B, Pell-Gregory)	25/25	2 ml Gengigel HyA gel (0.8%)	Intra-operative	1st, 3rd, 7th day	1 g amoxicillin 2 × 1 for 5 days; 550 mg naprexon sodium as needed	Edema, pain (VAS), trismus, number of pain killers
			25/25	Nothing				
Afat (2018)* RCT	38/22 18–30 ASA I Non-smoker	Unilaterally vertically impacted, partially erupted LM3 (class 2-B, Pell-Gregory)	20/20	L-PRF + HyA sponge	Intra-operative HyA sponge between 2 layers of L-PRF	6 h, 24 h, 2nd, 3rd, 4th, 5th, 6th, 7th day	“Standard postoperative medication”—no details reported	Edema, pain (VAS), trismus
			20/20	L-PRF				
			20/20	Nothing				
Bayoumi (2018) RCT, split-mouth	7/7 25.3 ± 3.1 Healthy NR	Bilateral symmetrical asymptomatic impacted LM3	14/14	0.33 ml HyadentBG HyA gel (0.2%) + Gelfoam	Intra-operative	2nd, 4th, 7th day	625 mg amoxicillin 3 × 1 for 5 days; 1 g paracetamol 3 × 1 for 5 days	Edema, pain (VAS), trismus
			14/14	Gelfoam				
Guazzo (2018) RCT	88/48 21.7 ± 2.4 ASA I-II < 10 cigarettes / day	Need of LM3 removal	65/56	2 ml Aminogam HyA gel + amino acid	Intra-operative	7th, 14th day	1 g amoxicillin 2 × 1 for 6 days; 1 g Paracetamol 3 × 1	Wound dehiscence, trismus, pus, pain on palpation, alveolitis, local lymphadenopathy, adverse reactions, number of pain killers
			71/50	Nothing				
Merchant (2018) RCT, split-mouth	15/15 25.8 ± 4.7 Healthy NR	Bilateral symmetrical impacted LM3	30/30	30 ml Kojimax Cosderma HyA spray (0.5%)	Spray 3 × /day for 7 days	2nd, 5th, 7th day	500 mg amoxicillin 3 × 1; 500 mg paracetamol/50 mg tramadol 2 × 1	Edema, pain (VAS), trismus
			30/30	Saline spray				
Afat (2019)* RCT	38/22 18–30 ASA I Non-smoker	Unilaterally vertically impacted, partially erupted LM3 (class 2-B, Pell-Gregory)	20/20	L-PRF + HyA sponge	Intra-operative HyA sponge between 2 layers of L-PRF	7th, 14th, 21st day	1 g amoxicillin 2 × 1; 500 mg paracetamol 3 × 1	Mucosa healing score, prolonged bleeding, alveolitis, wound infection
			20/20	L-PRF				
			20/20	nothing				
Munoz-Camara (2020) RCT	54/36 > 18 ASA I-II NR	Single asymptomatic impacted LM3	30/30	CHX + carbopol gel	Intra-operative	1st, 2nd, 3rd, 7th day	500 mg amoxicillin 3 × 1 for 7 days; 1 g paracetamol 3 × 1 for 4 days	Pain (VAS), trismus, alveolitis, wound infection, surgical difficulty, surgery duration
			30/30	1% HyA + carbopol gel				
			30/30	Carbopol gel				
Yang (2020) RCT	67/45 18–71 NR NR	Need of LM3 removal	56/52	0.25% HyA, Mucobarrier	Mouthwash	7th day	NR	Overall discomfort, pain (VAS), burning sensation, redness, swelling
			56/52	Aloclair				
Extraction socket								
Favia (2008) CT, split-mouth	NR NR NR NR	Bilateral extraction of molars	20/20	Aminogam HyA Gel (1.33%)	3 × /day	Up to 15 days	NR	Socket healing and keratinization
			20/20	Nothing				
Bayoumi (2015) RCT	NR 18–60 Healthy NR	Any permanent tooth	28/28	0.3 ml Hyadent HyA gel + Gelfoam	Intra-operative	1st, 2nd, 7th day	NR	Pain (VAS), alveolitis
			23/23	Gelfoam				
			57/57	Nothing				
Alcantara (2018) RCT, split-mouth	NR 18.7 ± 8.0 Healthy Non-smokers	Lower first premolar	16/16	1 ml Nikkol HyA gel (0.1%)	Intra-operative	30th, 90th day	750 mg paracetamol 4 × 1 for maximum 4 days	Alveolar dimensional changes, percentage of newly formed bone, mean fractal dimension
			16/16	Nothing				
Lorenz (2018) Prospective case series	11/10 51.4 Healthy Non-smokers and smokers	Any extraction socket with intact vestibular lamella for socket preservation	21/21	β-TCP, cellulose, HyA-IBS, collagen membrane	Intra-operative	4–6 months	Ibuprofen 400 mg	Amount of newly formed bone, vascularization, remaining biomaterial, connective tissue
Cocero (2019) RCT, split-mouth	NR NR Liver failure NR	Two symmetrical extraction sites	58/58	HyA gel	Intra-operative and 3 × /day	7th, 14th 21st day, until socket closure	NR	Alveolar diameter reduction, pain (VAS)
			58/58	Nothing				
Marin (2020) RCT, split-mouth	NR NR Type 2 diabetes Non-smokers	Two anterior teeth in the lower jaw	38/30	Gengigel Prof HyA gel (0.8%)	Intra-operative	1st, 2nd, 3rd, 5th, 10th, 15th, 20th, 25th day	NR	Wounds closure rate, wound healing score, pain (VAS)
			38/30	Nothing				
Mostafa (2021) RCT, split-mouth	9/6 18–44 Healthy Non-smokers	Two non-restorable single-rooted teeth	15/10	Gengigel HyA gel	Intra-operative and 3 × /day	1st, 5th, 10th day	NR	Socket length, socket healing scores, pain (VAS), soft tissue healing
			15/10	Nothing				
Eeckhout (2022) RCT	22/16 52.9 ± 15.8 Healthy Non-smokers	Need of extraction of 1 or 2 teeth in the aesthetic zone with > 50% buccal bone	23/23	Gengigel HyA gel (0.8%), BioOss collagen, Mucograft Seal	Gel 3 × /day for 7 days (i.e., not mixed with the other material in the socket)	7th, 21st day, 4th month	Amoxicillin 2 g for 4 days; ibuprofen 600 mg	Wound and alveolar dimensional changes, number of pain killers, pain (VAS), swelling, bleeding, socket healing
			23/23	BioOss collagen, Mucograft Seal				
Cosola (2022) RCT	20/20 46.5 ± 9.8 Healthy Non-smokers and smokers	Need of single tooth extraction	20/20	Aminogam HyA gel 0.2% CHX rinse, collagen sponge	Gel 1 × /day for 15 days (i.e., not with the collagen sponge into the socket)	7th, 14th, 30th, 60th day	NR	Pain (VAS), swelling, number of pain killers
			20/20	0.2% CHX rinse, collagen sponge				
Alveolar osteitis (AO)								
Dubovina (2016) RCT	24/36 NR NR NR	AO defined by Bloom et al. (2002)	10/10	HyA (0.2 ml Gengigel Prof, 0.8%) + irrigation	Intra-operative	Every 2nd day until absence of pain	No additional medication	Pain (VAS), pain irradiation, local lymphadenopathy, redness of gingiva, halitosis, number of visits
			10/10	HyA + ACA + irrigation				
			10/10	Alvogyl + irrigation				
			10/10	HyA + curettage				
			10/10	HyA + ACA + curettage				
			10/10	Alvogyl + curettage				
Suchanek (2019) Prospective case series	35/23 36.1 ± 12.2 NR ≤ 10 cigarettes/day	Any extraction site (including wisdom teeth) diagnosed with AO	58/50	Lyophilized water solution of 2.5% HyA, ODC, and calcium chloride	Sponge-like medical device inserted daily	Daily and 2 more days after resolving of pain	NR	Pain (VAS), adverse reactions

*ACA* aminocaproic acid, *ASA* American Society of Anesthesiologists, *BnzHCl* benzydamine hydrochloride, *β-TCP* beta-tricalcium phosphate, *CHX* chlorhexidine, *HyA* hyaluronic acid, *CT* controlled trial, *IBS* injectable bone substitute, *L-PRF* leukocyte- and platelet-rich fibrin, *NR* not reported, *ODC* octenidine dihydrochloride, *RCT* randomized controlled trial, *VAS* visual analogue scale

^*^Presumably same patient cohort/group

In the studies on LM3 extraction, the teeth were asymptomatic, predominantly vertically impacted or half impacted allowing primary wound closure after surgical removal. Half of the studies on extraction of regularly erupted teeth included only single rooted teeth (either anterior teeth or premolars), whereas the other half included either molars or any type of tooth. Both studies in the AO treatment group included all tooth types fulfilling the criteria of AO according to Blum et al. (2002) [57].

### Study intervention

In all preclinical trials, HyA was applied as a gel into the extraction socket directly after tooth removal either alone (*n* = 3) or in combination with an absorbable collagen sponge (*n* = 2) (Table 1).

In most clinical studies (*n* = 19) (Table 2), HyA was applied as a gel intra-operatively into the extraction socket or post-operatively at the extraction site either alone (*n* = 13) or with some carrier ((i.e., absorbable collagen sponge (*n* = 3), leukocyte- and platelet-rich fibrin (*n* = 2), or bone substitutes (*n* = 1)). In the remaining 3 clinical studies, HyA was either used as spray 3 times per day for 1 week (*n* = 2) or as mouthwash (*n* = 1).

### HyA information

In the 5 preclinical and 22 clinical studies included, 11 commercial, 2 self-made, and 2 of unknown origin HyA products were used (Table 3). In all preclinical studies (*n* = 5), HyA was applied as a gel, while in the clinical studies HyA was applied as gel (*n* = 15), spray (*n* = 2), mouthwash (*n* = 1), or combined with a sponge (*n* = 3) or bone substitute material (*n* = 1) during the fabrication process. The concentration of HyA varied from 0.2% in a spray, 0.25% in a mouthwash, up to 2.5% in a self-combined HyA sponge, while in 5 studies the concentration of HyA was not reported. The chemical form, i.e., non-cross-linked or cross-linked, was not reported in most of the studies (*n* = 16), whereas 10 studies used non-cross-linked HyA, and one study combined non- and cross-linked HyA.

**Table 3:** Overview of HyA products used in the preclinical and clinical trials

Product (Trade name)	Producer (Manufacturer, country)	HyA concentration (%)	Chemical form	Application form	Study (year)
Aminogam	Errecappa Euroterapici, Italy	1.33	Non-cross-linked	Gel	Favia (2008), Guazzo (2018), Cosola (2022)
Galena	Campinas, Brazil	1	NR	Gel	Sa (2013)
Gengigel	Farmalink, Turkey	0.2	Non-cross-linked	Spray	Koray (2014)
Gengigel	Ricerfarma, Italy	0.8	Non-cross-linked	Gel	Gocmen (2015, 2017), Dubovina (2016), Marin (2020), Eeckhout (2022)
	NR	NR	NR		Yilmaz (2017), Mostafa (2021)
Healon	Pharmacia & Upjohn, Sweden	1	NR	Gel	Kim (2016, 2019), Lee (2021)
Hyadent	BioScience, Germany	1.4	Non-cross-linked	Gel	Bayoumi (2015)
HyadentBG	BioScience, Germany	1.6 0.2	Cross-linked Non-cross-linked	Gel	Bayoumi (2018)
Hyalomatrix	Anika Therapeutics, USA	NR	NR	Sponge	Afat (2018, 2019)
Kojimax	Cosderma, India	0.5	NR	Spray	Merchant (2018)
Mucobarrier	NR	0.25	NR	Mouthwash	Yang (2020)
Nikkol	BS Pharma, Belo Horizonte, Brazil	1	NR	Gel	Mendes (2008), Alcantara (2018)
Purpose-made HyA product	Sigma-Aldrich Chemistry, Spain	1	NR	Gel	Munoz-Camara (2020)
Purpose-made HyA product	Contipro, Czech Republic	2.5	NR	Sponge	Suchanek (2019)
HyA-based injectable bone substitute material	Unknown	NR	NR	Injection	Lorenz (2018)
HyA gel	Unknown	NR	NR	Gel	Cocero (2019)

*HyA* hyaluronic acid, *NR* not reported

### Clinical setting and funding details

All preclinical trials were funded by independent single [32, 33, 58] or multiple research grants [30, 59].

In one clinical study, a multicenter study design was reported including 8 medical centers [56], whereas all other clinical studies were performed in a single department in a university setting. Eleven clinical studies did not report on funding sources, while in 9 clinical studies [35, 36, 42, 44, 45, 52, 53, 56, 60] the funding was provided by the department; however, in 3 out of these 9 studies, the HyA gel was provided by the manufacturer [46, 52, 53]. In a single study, the funding was provided by 3 different research foundations [48].

### Reported outcome variables and follow-up

In the preclinical studies, bone formation was assessed by different methods between 14 days and 3 months postoperatively. One study investigated in addition the level of bone morphogenetic protein-2 and osteopontin (Table 1). Furthermore, 4 studies recorded no side effect after HyA application, while one study did not report absences/presence of side effects.

In the clinical studies, the evaluated outcome parameters varied depending on treatment indication (Table 2). In the studies on surgical removal of LM3, presence of pain measured by visual analogue scale (VAS), swelling, and trismus were the outcome parameters most often evaluated. Other less frequently assessed parameters were presence/absence of prolonged bleeding, presence/absence of soft tissue dehiscence, speed of mucosal healing, rate of AO/wound infection, and laboratory markers of inflammation, oxidative stress, and wound healing. Among the studies on extraction of regularly erupted teeth, 3 publications used different socket-/soft tissue healing scores, 3 publications assessed the amount of newly formed bone and/or alveolar dimensional changes, 3 publications assessed pain, and one study assessed the rate of AO. Both studies on AO treatment focused on assessment of pain and adverse reactions. Most of the clinical studies recorded no side effects after local application of HyA, while 6 studies did not mention the absences/presence of side effects. A single study [37] applying 0.8% HyA gel after LM3 removal reported a significantly prolonged bleeding time after wound closure compared to the control group; however, as hemostasis was within a physiological timeframe, this was not considered an adverse event.

### Summary of the results of the individual studies

In all preclinical studies (Table 4), based on histologic, radiologic, or immunohistochemical analysis, the test groups with HyA showed significantly better results compared to the control group in at least one of the parameters regarding bone formation; this was independent of socket condition (healthy or infected) and type of control treatment.

**Table 4:** Results of histologic, radiographic, and/or immunohistochemical analyses after application of HyA in extraction socket models in preclinical trials

Study (year)	Intervention	HyA concentration	Evaluation time	Bone formation (Histologic^1^, radiologic^2^, and immunohistochemical analysis^3^)					
Extraction sockets in healthy rats									
Mendes (2008)		1%	21st day	Bone trabeculae (%)^1^					
				Apical third of socket			Medial third of socket		
	HyA			**71.6 ± 2.8**			**67.9 ± 2.6**		
	Carbopol*			–			–		
	Blood clot			60.6 ± 1.7			59.4 ± 1.5		
Extraction sockets in healthy and diabetic rats									
Sa (2013)		1%	14th day	Bone trabeculae (%)^1^					
				Apical third of socket			Medial third of socket		
	Carbopol (non-diabetic)			**40.6 ± 4.9**			**43.3 ± 8.4**		
	Carbopol (diabetic)			16.6 ± 7.2			5.8 ± 3.5		
	HyA (diabetic)			**34.9 ± 5.3**			**23.9 ± 4.3**		
	HyA + SWCNT (diabetic)			**38.2 ± 1.1**			**35.6 ± 6.3**		
Infected extraction sockets in dogs									
Kim (2016)		1%	3rd month	MB (%)^1^			Bone marrow (%)^1^		
	HyA			**63.3 ± 9.8**			**34.7 ± 8.9**		
	Blood clot			47.8 ± 6.6			50.5 ± 6.4		
Kim (2019)		1%	3rd month	Net area (%)^2^**		BV/TV (%)^2^		OCN^3^	
	ACS			− 6.5 ± 9.8		17.9 ± 6.0		83.0 ± 27.6	
	HyA + ACS			**11.7 ± 4.7**		20.1 ± 6.3		**319.0 ± 138.6**	
	rhBMP-2 + ACS			**15.9 ± 3.1**		20.1 ± 6.6		**281.7 ± 125.7**	
	Blood clot			− 10.7 ± 1.8		18.0 ± 6.6		88.7 ± 43.0	
Lee (2021)		1%	3rd month	MB (%)^1^	NFB (%)^1^	CT (%)^1^	RGP (%)^1^	BV/TV (%)^2^	BS/TV (%)^2^
	ACS			45.2 ± 3.1	7.5 ± 2.2	**17.1 ± 6.8**		36.0 ± 10.4	18.6 ± 4.1
	HyA + ACS			**64.7 ± 3.9**	**15.5 ± 2.4**	10.8 ± 4.9		**53.3 ± 7.4**	**22.7 ± 3.6**
	DBBM-C			41.9 ± 5.0	5.6 ± 1.4	**35.1 ± 10.5**	3.7 ± 1.4	38.2 ± 7.9	**24.7 ± 5.6**
	HyA + DBBM-C			**59.9 ± 5.4**	**11.3 ± 3.1**	12.3 ± 5.6	2.9 ± 2.0	**46.3 ± 13.0**	**24.7 ± 2.3**

*ACS* absorbable collagen sponge, *BS/TV* bone surface per tissue volume, *BV/TV* bone volume per tissue volume, *CT* connective tissue, *DBBM-C* deproteinized bovine bone mineral with collagen, *HyA* hyaluronic acid, *MB* mineralized bone, *NFB* newly formed bone, *OCN* osteocalcin, *RGP* residual graft particles, *rhBMP* recombinant human bone morphogenetic protein, *SWCNT* single-walled carbon nanotube, *µCT* micro computed tomography

All data are presented as mean ± standard deviation unless indicated otherwise. Bold numbers indicate statistical significance in comparison to the control group, except for the following studies: (1) In Sa (2013), bold numbers indicate statistical difference between the test groups and the diabetic control (Carbopol) group; (2) in Kim (2019), the “ACS” and “Blood clot” were considered control groups and bold numbers indicate statistical significance in comparison to these groups; and (3) in Lee (2021), bold numbers indicate statistical significance in inter-group comparison (i.e., comparing ACS with HyA + ACS as well as DBBM-C with HyA + DBBM-C)

^*^This specific group was evaluated at a different timepoint

^**^Net area indicates alveolar bone overgrowth (positive value) or alveolar bone destruction (negative value)

^1^Histologic assessment of bone formation

^2^Radiologic assessment of bone formation

^3^Immunohistochemical analysis of bone formation

In 4 out of 10 clinical studies on surgical LM3 removal (Table 5) reporting on pain, significant advantages for the test group using HyA, compared to the control group, were reported in at least one postoperative timepoint. Similarly, in 4 out of 7 studies and in 3 out of 9 studies reporting on swelling and trismus, respectively, significant advantages in favor of HyA application compared to the control group were reported. In 3 out of 4 studies reporting on soft tissue healing after extraction of regularly erupted teeth, significantly improved soft tissue healing after HyA application compared to the control group was recorded. Furthermore, one study reported improved bone formation after 30 days, one study reported a decreased reduction of alveolar diameter after up to 21 days, while 2 studies reported either no difference between the groups or significant disadvantages for the test group using HyA in terms of alveolar dimensional changes. Finally, pain perception was reported in 6 studies, but only 2 studies reported significant differences between the groups in favor of HyA application. One study assessing treatment of AO reported significantly lower postoperative pain after HyA application compared to the application of alvogyl; the second study had no control group.

**Table 5:**
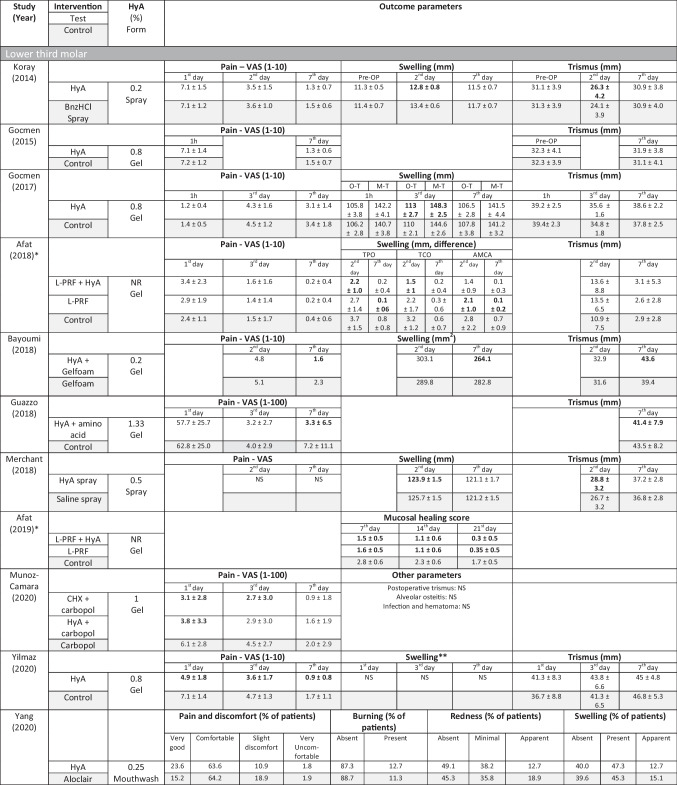

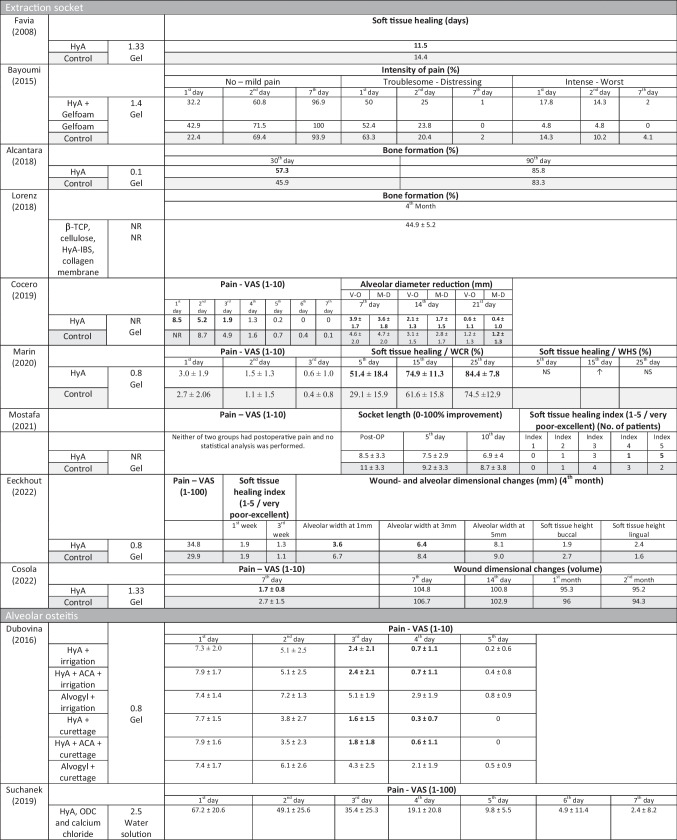
Results of clinical, laboratory, and radiographic analyses after application of HyA after (1) lower third molar removal, (2) extraction socket treatment other than LM3, and (3) treatment of alveolar osteitis

*ACA* aminocaproic acid, *AMCA* angulus mandibulae to lateral canthus, *ACS* absorbable collagen sponge, *CHX* chlorhexidine, *GSH* glutathione, *HyA* hyaluronic acid, *IBS* injectable bone substitute, *LPO* lipid peroxidation, *L-PRF* leucocyte- and platelet-rich fibrin, *M-*D mesio-distal, *M-T* meno-tragal distance, *NR* not reported, *NS* not significant, *O-T* oro-tragal distance, *ODC* octenidine dihydrochloride, *TCP* tragus to labial commissure, *TPO* tragus to pogonion, *VAS* visual analogue scale, *V–O* vestibulo-oral, *WCR* wound closure rate, *WHS* wound healing scale, *β-TCP* beta-tricalcium phosphate

Data are presented as mean ± standard deviation unless indicated otherwise. Bold numbers indicate statistical significance in comparison to the control group, except for Dubovina (2016) where bold numbers indicate statistical significance in inter-group comparison (i.e., comparing HyA + irrigation vs. Alvogyl + irrigation; HyA + ACA + irrigation vs. Alvogyl + irrigation; HyA + curettage vs. Alvogyl + curettage, and HyA + ACA + curettage vs. Alvogyl + curettage)

^*^Presumably same patient cohort/group

^**^swelling measurements were obtained from 7 different distances but are for simplicity of the table not presented in detail herein

↑Significantly higher/better

### *Synthesis of results*

#### Preclinical studies—bone volume per tissue volume in preclinical trials

**Fig. 1:**
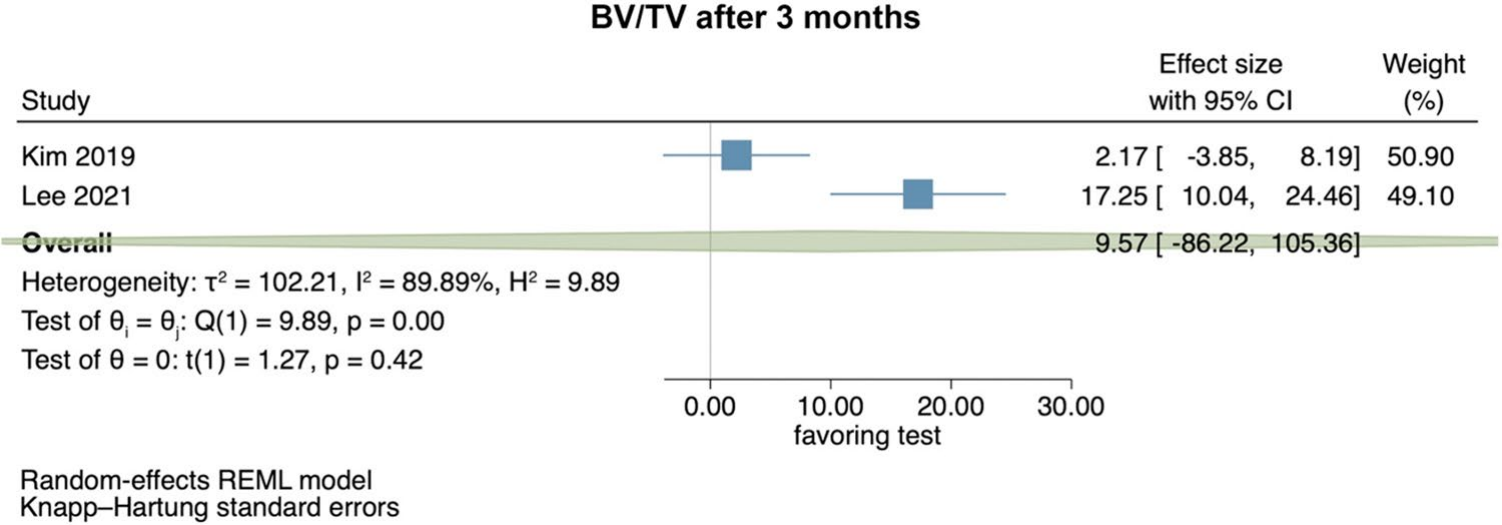
Forest plot on the effect size of HyA application (test) on BV/TV after 3 months compared to the control group in preclinical trials

Two preclinical studies provided data to summarize radiographically assessed BV/TV 3 months postoperatively [33, 58] (Fig. 1). The studies compared the application of HyA in combination with an absorbable collagen sponge versus the absorbable collagen sponge. Overall, no significant difference between the groups was identified (effect size: 9.57; 95% CI: − 86.22 to 105.36; *p* = 0.42), but statistical heterogeneity among the studies was significant (*I*^2^ = 89.89%; *p* < 0.01).

#### Clinical studies—evaluation of pain 2–3 and 7 days after surgical LM3 removal

**Fig. 2:**
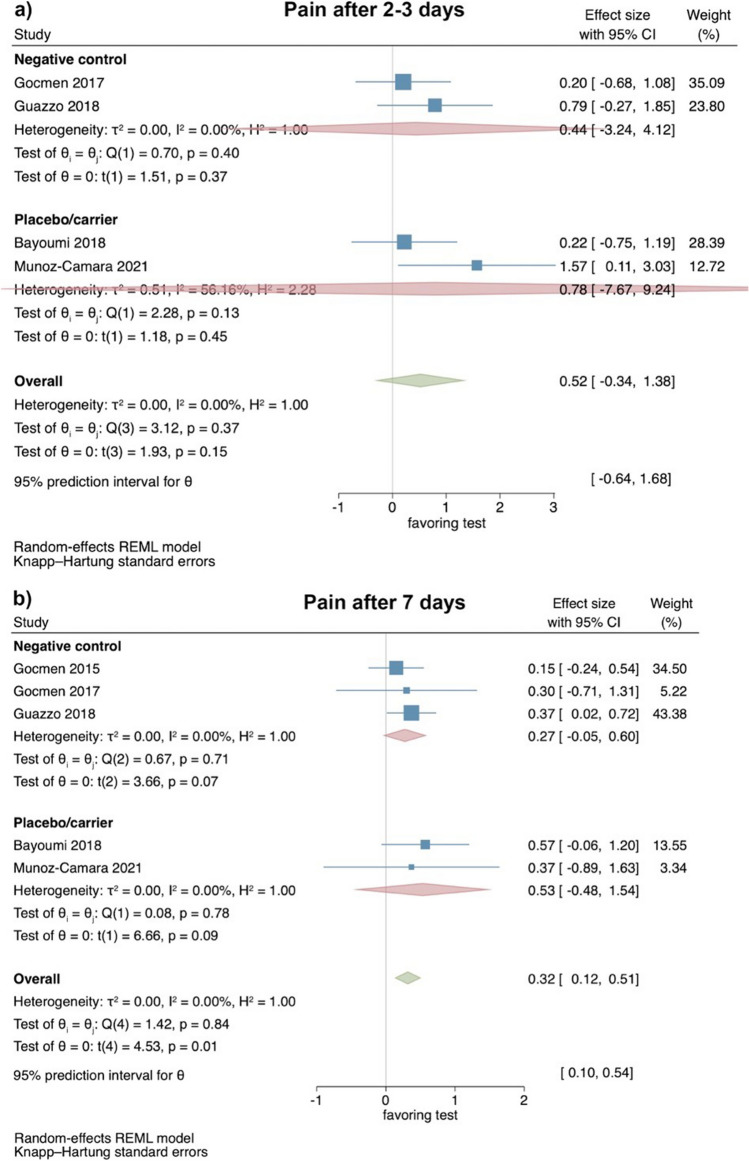
Forest plot on the effect size of HyA application (test) on pain after surgical LM3 removal 2–3 days (**a**) and 7 days (**b**) postoperatively

Based on the results of 4 RCTs [37, 39, 40, 61], perception of pain showed no statistical significant differences between test and control groups 2–3 days postoperatively (effect size: 0.52; 95% CI: − 0.34–1.38; *p* = 0.15), without statistical heterogeneity among the studies (*I*^2^ = 0.00%; *p* = 0.37). Separate analyses with 2 studies each comparing HyA with a negative control group (effect size: 0.44; 95% CI: − 3.24–4.12; *p* = 0.37) and HyA with a placebo/carrier group (effect size: 0.78; 95% CI: − 7.67–9.24; *p* = 0.45) lacked also statistical significance (Fig. 2a).

Based on the results of 5 RCTs [36, 37, 39, 40, 61], perception of pain 7 days postoperatively was significantly lower in the test groups applying HyA (effect size: 0.32; 95% CI: 0.12–0.51; *p* = 0.01), without statistical heterogeneity among the studies (*I*^2^ = 0.00%; *p* = 0.84). However, the separate analyses lacked statistical significance for the comparison HyA with a negative control group (3 studies; effect size: 0.27; 95% CI: − 0.05–0.60; *p* = 0.07) and for the comparison HyA with a placebo/carrier group (2 studies; effect size: 0.53; 95% CI: − 0.48–1.54; *p* = 0.09; Fig. 2b).

#### Clinical studies—evaluation of swelling 2–3 and 7 days after surgical LM3 removal

**Fig. 3:**
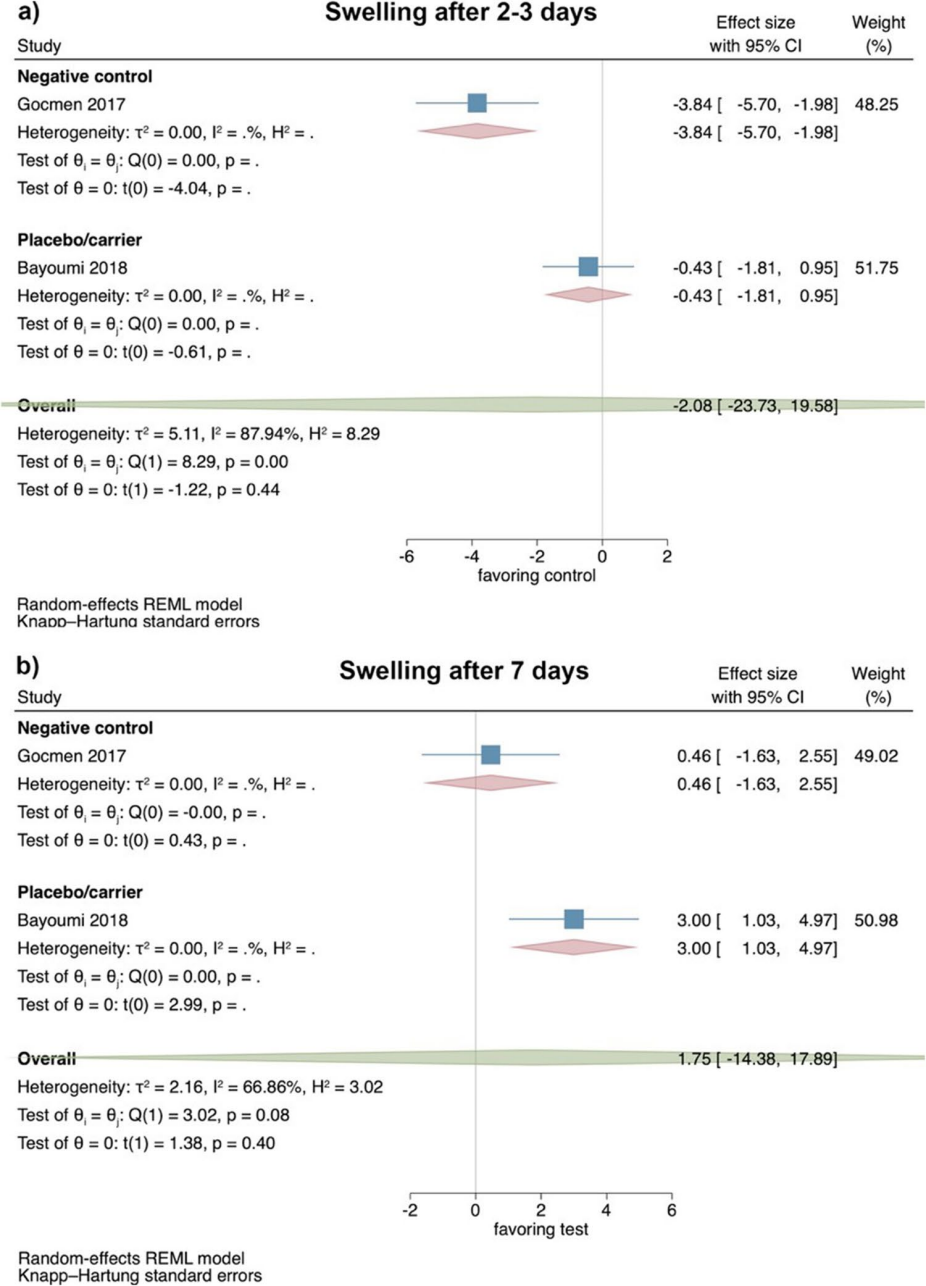
Forest plot on the effect size of HyA application (test) on swelling after surgical LM3 removal 2–3 days (**a**) and 7 days (**b**) postoperatively. The values of both studies are based on a length measurement (i.e., from the ear to the corner of the mouth in mm); please note that the original data set has been provided by Bayoumi et al.

Based on the results of 2 RCT [37, 39], the extent of swelling 2–3 days postoperatively showed no significant difference between test and control groups (effect size: − 2.08; 95% CI: − 23.73–19.58; *p* = 0.44); however, statistical heterogeneity among the studies was significant (*I*^2^ = 87.94%; *p* < 0.01; Fig. 3a).

Similarly, the extent of swelling 7 days postoperatively also showed no significant difference between test and control groups (effect size: 1.75; 95% CI: − 14.38–17.89; *p* = 0.40), and statistical heterogeneity among the studies was again significant (*I*^2^ = 66.86%; *p* = 0.08; Fig. 3b). No separate analysis for the comparison HyA with either a negative control or placebo/carrier group was possible due to the limited number of studies.

#### Clinical studies—evaluation of trismus 2–3 and 7 days after surgical LM3 removal

**Fig. 4:**
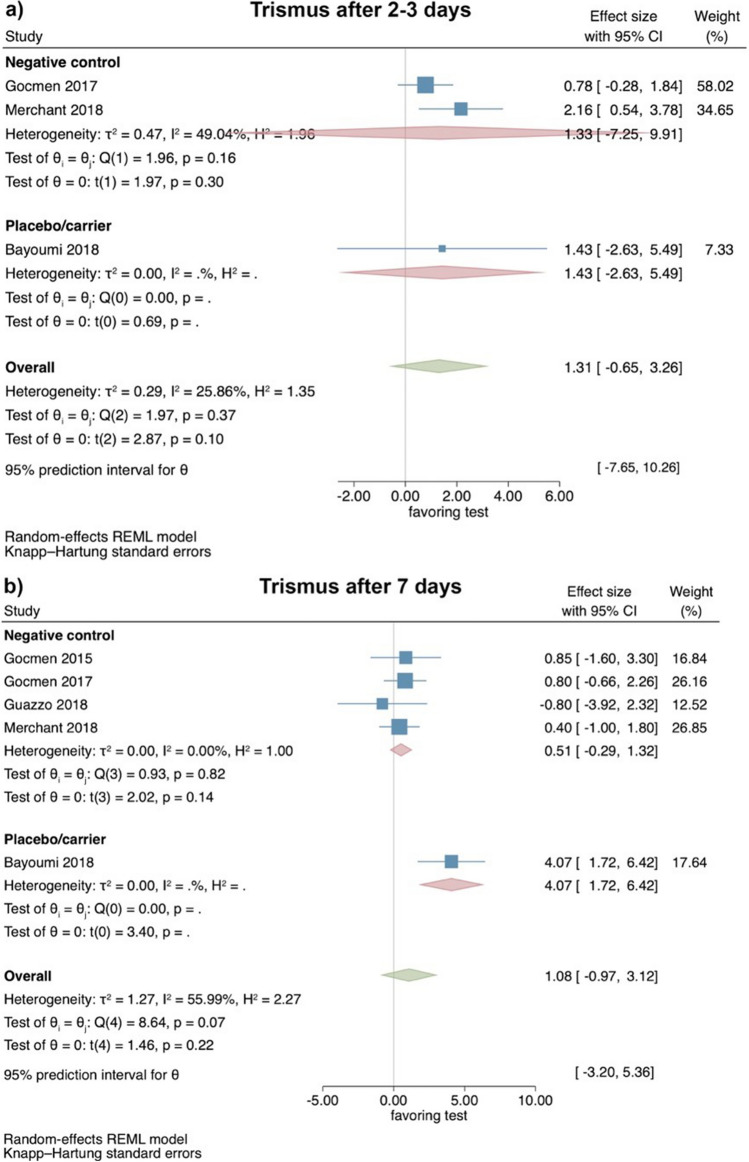
Forest plot on the effect size of HyA application (test) on trismus after surgical LM3 removal 2–3 days (**a**) and 7 days (**b**) postoperatively

Based on the results of 3 RCTs [37, 39, 41], trismus showed 2–3 days postoperatively no significant differences between test and control groups (effect size: 1.31; 95% CI: − 0.65–3.26; *p* = 0.10), without statistical heterogeneity among the studies (*I*^2^ = 25.86%; *p* = 0.37). The separate analyses also lacked statistical significance for the comparison HyA with a negative control group (2 studies; effect size: 1.33; 95% CI: − 7.25–9.91; *p* = 0.30), while only a single study was available for the comparison HyA with a placebo/carrier group (Fig. 4a).

Based on the results of 5 RCTs [36, 37, 39–41], trismus showed no significant difference between test and control groups 7 days postoperatively (effect size: 1.08; 95% CI: − 0.97–3.12; *p* = 0.22); however, statistical heterogeneity among the studies was significant (*I*^2^ = 55.99%; *p* = 0.07). The separate analyses also showed no significant difference between HyA and a negative control group (4 studies; effect size: 0.51; 95% CI: − 0.29–1.32; *p* = 0.14), while only a single study was available for the comparison HyA with a placebo/carrier group (Fig. 4b).

### Risk of bias assessment

Among the preclinical studies, the quality score ranged between 20 and 40% (Appendix 4); only reporting of baseline characteristics and other sources of bias were judged in all studies as low risk of bias.

The included RCT were either judged as having some concerns (*n* = 13) or low risk of bias (*n* = 5) (Appendix 5). None of the RCT deviated from the intended intervention, 5 RCTs were judged as having some concerns in the randomization process, and approximately half of studies were judged as having some concerns in their reporting on missing outcome data, measurement of the outcome, and selection of the reported results. Most of the non-randomized studies were judged as having a low risk of bias (*n* = 3), whereas one study was judged as having some concerns (Appendix 6).

### *Quality of evidence (GRADE)*

For the results of the meta-analysis including 2 preclinical trials, the certainty of evidence for the outcome parameter BV/TV after 3 months was rated low (Appendix 8a).

The certainty of evidence obtained from meta-analyses including clinical trials was judged as moderate for pain perception and trismus and as low for the swelling assessment (Appendix 8b).

## Discussion

HyA has been shown to possess anti-inflammatory, anti-edematous, osteoinductive, and pro-angiogenetic properties; thus, it appears that HyA improves wound healing [62–65]. The present systematic review aimed to provide a comprehensive assessment of all available evidence (i.e., preclinical and clinical) on the effect of HyA application in connection with tooth extraction. Overall, it seems that HyA application in connection to surgical LM3 removal may have a positive effect in pain reduction during the first post-operative week. Specifically, meta-analysis of 5 clinical studies showed that local (intra-surgical) application of HyA gel was associated with a statistically significantly reduced perception of pain 7 days postoperatively compared to the control group with either no additional wound manipulation or the application of a placebo/carrier. HyA application did not seem to have any impact on other often appearing complications after LM3 removal (i.e., swelling and trismus) or in connection with non-surgical extraction of normally erupted teeth.

This positive effect of intra-surgical application of HyA on pain perception within the first post-operative week of LM3 removal adds on the results of a previous systematic review, which also assessed the possible benefit of HyA in the same indication [17]. Specifically, based on a different study selection, HyA application significantly reduced pain on both the 3rd and 7th postoperative day [17]. Apparently, the positive effect of HyA in the very early post-operative days observed in that review was not seen in the present meta-analysis, due to the increased information provided by 2 additional studies [39, 61] included herein and due to the exclusion of a non-randomized study, which strongly favored the HyA test group [44]. A positive effect of HyA in terms of reduced pain perception can be partly explained by its modulating effect on the inflammatory response at the surgical site. It has been previously demonstrated that HyA can downregulate the production and expression of prostaglandin E_2_, bradykinin, and substance P, which are all involved in pain transmission and sensation [66]. Nevertheless, any potential positive effect of HyA on the local inflammatory response does not necessarily translate in less swelling and/or trismus in the clinic, since both the analyses included herein and those in the above-mentioned review failed to indicate any differences between test and control groups regarding these aspects. However, these results should be interpreted with care due to the small number of original studies and the lack of standardization in the methods assessing facial swelling as well as in the intervention per se. For example, the included studies seldomly provided information on the level of surgical difficulty and/or applied flap design, aspects which may affect the outcome parameters [67]. Moreover, the lack of any significant positive effect of HyA in pain perception in non-surgical extraction of regularly erupted teeth, seen in most studies (4 out 5) included in this review, should not be interpreted as lack of action of HyA per se. It may be due differences in the healing mode, i.e., “closed” after surgical LM3 removal versus “open” after extraction of regularly erupted teeth, where the lack of primary closure and of any carrier may have resulted in a fast wash-out of HyA. Whether the application of HyA in a carrier could improve its action, is difficult to assess, as this was used only in a single study that failed to show any differences [47]. Nevertheless, it should also be kept in mind that in most cases, uncomplicated tooth extraction is associated with low levels of pain, and thus any possible positive effect of HyA may be difficult to capture. In fact, in the only comparative study on AO management included in this review, significantly reduced pain postoperatively in the groups receiving HyA (with no primary closure and no use of a carrier) was reported.

Some of the studies on healing after extraction of regularly erupted teeth, included in this review, also assessed the possible impact of HyA application on soft and hard tissue healing. In 3 out of 4 studies assessing soft tissue healing, a positive effect of HyA was reported based on the time until and/or percentage of socket closure, as well as based on scores for judging soft tissue healing. In contrast, in 3 comparative studies, intra- or post-operative use of HyA gel did not have any positive effect in terms of alveolar dimensional changes compared to no HyA application, after a follow-up time of 3 to 4 months [48, 52, 53]. In fact, in one of the studies [52], where following ridge preservation with socket grafting with collagen-enriched, deproteinized bovine bone mineral and socket sealing by means of a collagen matrix surgical therapy, HyA gel was applied onto the collagen matrix three times per day for 1 week, significantly more horizontal bone loss at the coronal aspect of the extraction sockets was observed. These findings on lack of a positive effect of HyA on bone may appear somehow in contrast with the findings reported in the preclinical studies included herein. In the 2 studies reporting on healing of non-infected extraction sockets in either healthy [30] or diabetic [31] rats, HyA application significantly enhanced bone healing compared to the control group. Similarly, in 3 out of 3 dog studies reporting on healing of infected extraction sockets, HyA application either alone [32] or with a collagen sponge [33, 58] or deproteinized bovine bone mineral with collagen as carrier [58] enhanced bone healing. It is important to mention, however, that this positive effect of HyA on bone healing was not shown in the only meta-analysis possible herein regarding BV/TV, probably due to the fact that both studies used a late healing time for this particular animal model; i.e., bone healing inside an extraction socket in the dog is rather advanced after 3 months, even without any treatment [68]. Noteworthy, BV/TV in the HyA group was similar to that in another test group, treated with recombinant human bone morphogenetic protein-2 (rhBMP-2), a known very potent bone enhancing agent [33, 58]. Furthermore, such positive effects of HyA on bone healing have also been shown in other preclinical studies, using critical size defect models [13, 14]. In perspective, no study on surgical removal of LM3 assessed the healing outcome at the distal aspect of the lower second molar, a site that is often associated with a deep periodontal defect after extraction of impacted LM3 [5].

This review tried also to identify whether application of HyA may reduce the rate of AO after tooth extraction; however, there was limited reporting on this complication in the studies. In this context, application of HyA is in general considered safe and with no side effects; however, it must be mentioned that HyA may lead to significant adverse events in case it is applied (injected) within the tissues [69]. Herein, only a single study [37] reported a prolonged bleeding time after wound closure compared to the control group; however, hemostasis was judged to be within a physiological timeframe and therefore not considered an adverse event. All other studies included in this review did not mention any side effects or complications after HyA application. Besides the fact that HyA is safe to apply in connection with surgical LM3 or non-surgical tooth extraction, no conclusions can be made regarding the most efficient HyA formulation (e.g., low vs. high concentration, non-cross-linked vs. cross-linked, gel vs. spray) or application mode (e.g., with vs. without a carrier, frequency), and thus no clear recommendation can be provided.

Altogether, only a limited number of well-designed, randomized preclinical and clinical trials could be identified herein and combined in a meta-analysis. Moreover, as outlined above, there is a lack of consensus and information on HyA product details, but also on the surgical details (e.g., level of surgical difficulty or flap design). These limitations resulted in an overall low to moderate certainty of evidence. In future studies, a better and more standardized reporting on HyA product details, dosage, and application, and longer follow-up times should be implemented to allow for a more complete evaluation of the potential of HyA use in connection with tooth extraction. In addition, future updated systematic reviews including a larger number of studies should also consider in the meta-analyses a comparison between studies with parallel arms and studies in split-mouth design. This would be specifically of interest for parameters such as pain perception, something not feasible herein due to the very limited number of split-mouth studies [39, 41].

## Conclusion

The results of the present systematic review and meta-analyses showed that intra-surgical application of HyA in connection with surgical LM3 removal resulted in significant reduction in pain perception 7 days postoperatively, while early post-operative pain, trismus, and extent of swelling were unaffected. Furthermore, it seems that HyA application may have a positive effect on soft tissue healing after non-surgical extraction of normally erupted teeth, but it seems not to reduce post-extraction alveolar ridge modeling even though evidence from preclinical studies indicated that HyA may enhance bone formation.

### Supplementary Information

Below is the link to the electronic supplementary material.

Supplementary file1 (DOCX 100 KB)

## Data Availability

Data are available from the authors upon reasonable request.
