# Supplementary material for: Hyaluronic acid in tooth extraction: a systematic review and meta-analysis of preclinical and clinical trials
Source: Clin Oral Investig. 2023 Nov 15;27(12):7209–29. doi: 10.1007/s00784-023-05227-4 (PMC10713798; doi:10.1007/s00784-023-05227-4)
Supplement: Supplementary file 1 — Supplementary file1 (DOCX 100 KB) [file 784_2023_5227_MOESM1_ESM.docx]

# Appendix legends

Appendix 1. PRISMA 2020 Checklist.

Appendix 2. Details on the literature search.

Appendix 3. Flowchart of the literature search.

Appendix 4. Risk of bias assessment of the preclinical trials (SYRCLE’s RoB tool).

Appendix 5. Risk of bias assessment of the randomized controlled clinical trials (Cochrane Collaboration’s RoB 2.0 tool).

Appendix 6. Risk of bias assessment of non-randomized clinical trials (ROBINS-I tool).

Appendix 7. Reasons for exclusion of 6 full-text articles.

Appendix 8. Quality of Evidence (GRADE) / Summary of Findings Tables for (a) preclinical and (b) clinical trials.

| **Section and Topic** | **Item #** | **Checklist item** | **Location where item is reported** |
| --- | --- | --- | --- |
| **TITLE** | | |  |
| Title | 1 | Identify the report as a systematic review. | Front page |
| **ABSTRACT** | | |  |
| Abstract | 2 | See the PRISMA 2020 for Abstracts checklist. | Page 2. |
| **INTRODUCTION** | | |  |
| Rationale | 3 | Describe the rationale for the review in the context of existing knowledge. | Chapter 2., page 3-4. |
| Objectives | 4 | Provide an explicit statement of the objective(s) or question(s) the review addresses. | Chapter 2., page 3-4. |
| **METHODS** | | |  |
| Eligibility criteria | 5 | Specify the inclusion and exclusion criteria for the review and how studies were grouped for the syntheses. | Subheading 3.2; page 4. |
| Information sources | 6 | Specify all databases, registers, websites, organisations, reference lists and other sources searched or consulted to identify studies. Specify the date when each source was last searched or consulted. | Subheading 3.2; page 4. |
| Search strategy | 7 | Present the full search strategies for all databases, registers and websites, including any filters and limits used. | Appendix 2. |
| Selection process | 8 | Specify the methods used to decide whether a study met the inclusion criteria of the review, including how many reviewers screened each record and each report retrieved, whether they worked independently, and if applicable, details of automation tools used in the process. | Subheading 3.3; page 4-5. |
| Data collection process | 9 | Specify the methods used to collect data from reports, including how many reviewers collected data from each report, whether they worked independently, any processes for obtaining or confirming data from study investigators, and if applicable, details of automation tools used in the process. | Subheading 3.3; page 4-5. |
| Data items | 10a | List and define all outcomes for which data were sought. Specify whether all results that were compatible with each outcome domain in each study were sought (e.g. for all measures, time points, analyses), and if not, the methods used to decide which results to collect. | Table 1.,2.,4., and 5. |
|  | 10b | List and define all other variables for which data were sought (e.g. participant and intervention characteristics, funding sources). Describe any assumptions made about any missing or unclear information. | Table 1.,2.,4., and 5. |
| Study risk of bias assessment | 11 | Specify the methods used to assess risk of bias in the included studies, including details of the tool(s) used, how many reviewers assessed each study and whether they worked independently, and if applicable, details of automation tools used in the process. | Subheading 3.4; page 5. |
| Effect measures | 12 | Specify for each outcome the effect measure(s) (e.g. risk ratio, mean difference) used in the synthesis or presentation of results. | Subheading 3.5; page 5-6. |
| Synthesis methods | 13a | Describe the processes used to decide which studies were eligible for each synthesis (e.g. tabulating the study intervention characteristics and comparing against the planned groups for each synthesis (item #5)). | Subheading 3.5; page 5-6. |
|  | 13b | Describe any methods required to prepare the data for presentation or synthesis, such as handling of missing summary statistics, or data conversions. | Subheading 3.5; page 5-6. |
|  | 13c | Describe any methods used to tabulate or visually display results of individual studies and syntheses. | Table 1.,2.,4., and 5. |
|  | 13d | Describe any methods used to synthesize results and provide a rationale for the choice(s). If meta-analysis was performed, describe the model(s), method(s) to identify the presence and extent of statistical heterogeneity, and software package(s) used. | Subheading 3.5; page 5-6. |
|  | 13e | Describe any methods used to explore possible causes of heterogeneity among study results (e.g. subgroup analysis, meta-regression). | Subheading 3.5; page 5-6. |
|  | 13f | Describe any sensitivity analyses conducted to assess robustness of the synthesized results. | Subheading 3.5; page 5-6. |
| Reporting bias assessment | 14 | Describe any methods used to assess risk of bias due to missing results in a synthesis (arising from reporting biases). | Subheading 3.4; page 5. |
| Certainty assessment | 15 | Describe any methods used to assess certainty (or confidence) in the body of evidence for an outcome. | Subheading 3.5; page 5-6. |
| **RESULTS** | | |  |
| Study selection | 16a | Describe the results of the search and selection process, from the number of records identified in the search to the number of studies included in the review, ideally using a flow diagram. | Appendix 3. |
|  | 16b | Cite studies that might appear to meet the inclusion criteria, but which were excluded, and explain why they were excluded. | Subheading 4.1; page 6. |
| Study characteristics | 17 | Cite each included study and present its characteristics. | Table 1.,2.,4., and 5. |
| Risk of bias in studies | 18 | Present assessments of risk of bias for each included study. | Subheading 4.8; page 11. |
| Results of individual studies | 19 | For all outcomes, present, for each study: (a) summary statistics for each group (where appropriate) and (b) an effect estimate and its precision (e.g. confidence/credible interval), ideally using structured tables or plots. | Subheading 4.9; page 11. |
| Results of syntheses | 20a | For each synthesis, briefly summarise the characteristics and risk of bias among contributing studies. | Subheading 4.7; page 9-11; figure 1.,2.,3., and 4. |
|  | 20b | Present results of all statistical syntheses conducted. If meta-analysis was done, present for each the summary estimate and its precision (e.g. confidence/credible interval) and measures of statistical heterogeneity. If comparing groups, describe the direction of the effect. | Subheading 4.7; page 9-11; figure 1.,2.,3., and 4. |
|  | 20c | Present results of all investigations of possible causes of heterogeneity among study results. | Subheading 4.7; page 9-11; figure 1.,2.,3., and 4. |
|  | 20d | Present results of all sensitivity analyses conducted to assess the robustness of the synthesized results. | Subheading 4.7; page 9-11; figure 1.,2.,3., and 4. |
| Reporting biases | 21 | Present assessments of risk of bias due to missing results (arising from reporting biases) for each synthesis assessed. | Appendix 4., and 5. |
| Certainty of evidence | 22 | Present assessments of certainty (or confidence) in the body of evidence for each outcome assessed. | Subheading 4.7; page 9-11; figure 1.,2.,3., and 4. |
| **DISCUSSION** | | |  |
| Discussion | 23a | Provide a general interpretation of the results in the context of other evidence. | Heading 5., page 11-12. |
|  | 23b | Discuss any limitations of the evidence included in the review. | Heading 5., page 13-14. |
|  | 23c | Discuss any limitations of the review processes used. | Heading 5., page 13-14. |
|  | 23d | Discuss implications of the results for practice, policy, and future research. | Heading 6., page 14. |
| **OTHER INFORMATION** | | |  |
| Registration and protocol | 24a | Provide registration information for the review, including register name and registration number, or state that the review was not registered. | Subheading 3.1; page 4. |
|  | 24b | Indicate where the review protocol can be accessed, or state that a protocol was not prepared. |  |
|  | 24c | Describe and explain any amendments to information provided at registration or in the protocol. |  |
| Support | 25 | Describe sources of financial or non-financial support for the review, and the role of the funders or sponsors in the review. | Title page |
| Competing interests | 26 | Declare any competing interests of review authors. | Title page |
| Availability of data, code and other materials | 27 | Report which of the following are publicly available and where they can be found: template data collection forms; data extracted from included studies; data used for all analyses; analytic code; any other materials used in the review. |  |

*From:*  Page MJ, McKenzie JE, Bossuyt PM, Boutron I, Hoffmann TC, Mulrow CD, et al. The PRISMA 2020 statement: an updated guideline for reporting systematic reviews. BMJ 2021;372:n71. doi: 10.1136/bmj.n71

For more information, visit: http://www.prisma-statement.org

*Appendix 2.* Details on the literature search.

The literature search was performed in 3 databases (i.e., Ovid (MEDLINE and CENTRAL), EMBASE, Pubmed) and the last search was performed on 7^th^ of April 2022 (no date restriction was applied). The search on Ovid was performed as follows:

1. tooth extraction
2. ((tooth or teeth or dental* or third molar* adj5 (extract* or remov* or postextract* or postremov* or post operativ* or postoperativ* or postsurg* or post surg*)).ti,ab,kf.
3. ((socket* adj5 (extract* or remov* or postextract* or postremov* or post operativ* or postoperativ* or postsurg* or post surg*)).ti,ab,kf. and (dent* or tooth or teeth).mp.
4. hyaluronic acid or (hyaluron* adj4 (acid or sodium)).ti,ab,kf. or hyaluronan*.ti,ab,kf.

The asterisk (*) was used as a truncation symbol, and “adj” represented the number of words allowed to be in-between the keywords (e.g., “adj5” allowed 5 words in-between the keywords). Abbreviation “mp” stands for title (“ti”), abstract (“ab”), original title (“kf”), name of substance word, subject heading word, floating sub-heading word, keyword heading word, organism supplementary concept word, protocol supplementary concept word, rare disease supplementary concept word, unique identifier, and synonyms. The same keywords but adjusted to suit database specific criteria were used for the other 2 sources (EMBASE and Pubmed). Moreover, grey literature was browsed, and screening of the reference lists of available reviews and included studies was conducted. Finally, a forward search via Science Citation Index with the included publications was performed.

*Appendix 3.* Flowchart of the literature search.

Records excluded
(n = 57)

Records screened
(n = 90)

Full-text articles assessed for eligibility
(n = 33)

Records after duplicates removed
(n = 90)

Additional records identified through other sources
(n = 4)

Records identified through database searching
(n = 143)

## Identification

## Screening

Full-text articles excluded
(n = 6)*

## Eligibility

Studies included in quantitative synthesis:

Pre-clinical (n = 2)
Clinical (n = 6)

Studies included in qualitative synthesis:

Pre-clinical (n = 5)
Clinical (n = 22)

## Included

* Reasons for exclusion of 6 full-text articles and the reference list are provided in *Appendix 7*.

*Appendix 4.* Risk of bias assessment of the preclinical trials (SYRCLE’s RoB tool).

| **Study** | **Study design** | **Sequence generation** | **Baseline characteristics** | **Allocation concealment** | **Random housing** | **Blinding caregivers or researchers** | **Random outcome assessment** | **Blinding outcome assessor** | **Incomplete outcome data** | **Selective outcome reporting** | **Other sources of bias** | **Quality Score (%)** |
| --- | --- | --- | --- | --- | --- | --- | --- | --- | --- | --- | --- | --- |
| Mendes (2008) | RCT | 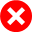 | 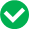 | 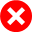 | 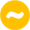 | 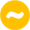 | 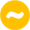 | 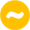 | 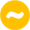 | 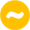 | 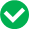 | 20 |
| Sa (2013) | CT | 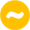 | 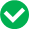 | 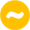 | 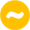 | 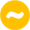 | 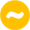 | 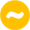 | 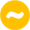 | 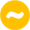 | 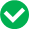 | 20 |
| Kim (2016) | RCT | 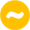 | 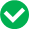 | 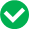 | 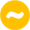 | 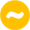 | 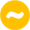 | 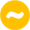 | 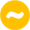 | 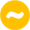 | 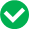 | 30 |
| Kim (2019) | RCT | 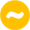 | 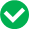 | 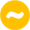 | 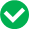 | 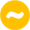 | 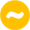 | 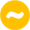 | 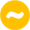 | 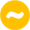 | 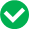 | 30 |
| Lee (2021) | RCT | 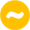 | 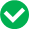 | 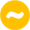 | 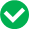 | 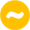 | 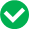 | 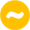 | 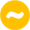 | 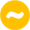 | 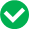 | 40 |

Extraction socket

Compromised healing (Diabetes mellitus)

Infected extraction socket


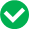
 Low
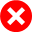
 High
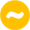
 Unclear risk of bias

*Appendix 5.* Risk of bias assessment of the randomized controlled clinical trials (Cochrane Collaboration’s RoB 2.0 tool).

| **Study** | **Randomization process** | **Deviations from intended interventions** | **Missing outcome data** | **Measurement of the outcome** | **Selection of the reported result** | **Overall** |
| --- | --- | --- | --- | --- | --- | --- |
| Koray (2014) | 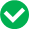 | 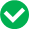 | 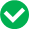 | 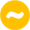 | 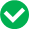 | 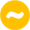 |
| Gocmen (2015) | 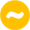 | 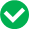 | 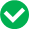 | 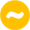 | 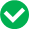 | 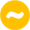 |
| Gocmen (2017) | 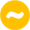 | 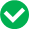 | 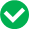 | 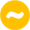 | 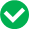 | 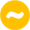 |
| Afat (2018) | 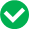 | 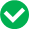 | 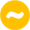 | 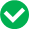 | 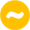 | 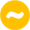 |
| Bayoumi (2018) | 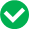 | 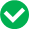 | 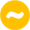 | 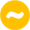 | 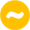 | 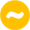 |
| Guazzo (2018) | 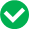 | 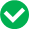 | 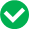 | 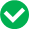 | 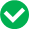 | 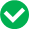 |
| Merchant (2018) | 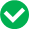 | 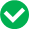 | 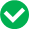 | 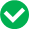 | 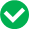 | 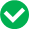 |
| Afat (2019) | 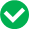 | 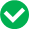 | 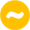 | 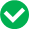 | 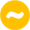 |  |
| Munoz-Camara (2020) |  |  |  |  |  |  |
| Yang (2020) |  |  |  |  |  |  |
| Bayoumi (2015) |  |  |  |  |  |  |
| Alcantara (2018) |  |  |  |  |  |  |
| Cocero (2019) |  |  |  |  |  |  |
| Marin (2020) |  |  |  |  |  |  |
| Mostafa (2021) |  |  |  |  |  |  |
| Eeckhout (2022) |  |  |  |  |  |  |
| Cosola (2022) |  |  |  |  |  |  |
| Dubovina (2016) |  |  |  |  |  |  |

Lower third molar

Extraction socket

Alveolar osteitis

Low risk of bias High risk of bias Some concerns

*Appendix 6.* Risk of bias assessment of the non-randomized clinical trials (ROBINS-I tool).

| **Study** | **Confounding** | **Selection of participants** | **Classification of interventions** | **Deviations from intended interventions** | **Missing outcome data** | **Measurement of the outcome** | **Selection of the reported result** | **Overall** |
| --- | --- | --- | --- | --- | --- | --- | --- | --- |
| Yilmaz (2017) | low | low | low | low | low | low | low |  |
| Favia (2008) | NI | moderate | moderate | NI | NI | moderate | moderate |  |
| Lorenz (2018) | low | low | low | low | low | low | low |  |
| Suchanek (2019) | low | low | low | low | low | low | low |  |

Lower third molar

Extraction socket

Alveolar osteitis

Overall risk of bias judgement: Low risk of bias High risk of bias Some concerns

NI- no information

*Appendix 7.* Reasons for exclusion of 6 studies after full-text analysis.

| Author (Year) | Title | Reason for exclusion |
| --- | --- | --- |
| Canciani (2021) | Effects of vitamin and amino acid-enriched hyaluronic acid gel on the healing of oral mucosa: in vivo and in vitro study. | Treatment modality not meeting the inclusion criteria |
| Puspitaningrum (2021) | Core-shell nanoparticles epigallocatechin-3-gallate and chitosan/hyaluronic-acid as a socket preservation for dental implant. | No full-text available |
| Kapitan (2021) | Initial Observation of Factors Interfering with the Treatment of Alveolar Osteitis Using Hyaluronic Acid with Octenidine-A Series of Case Reports. | < 10 patients |
| Martins-Junior (2020) | Carbon nanotubes functionalized with sodium hyaluronate: Sterilization, osteogenic capacity and renal function analysis. | Treatment modality not meeting the inclusion criteria |
| Catanzano (2018) | Composite Alginate-Hyaluronan Sponges for the Delivery of Tranexamic Acid in Postextractive Alveolar Wounds. | Study design not meeting the inclusion criteria |
| Mendes (2010) | Effects of single wall carbon nanotubes and its functionalization with sodium hyaluronate on bone repair. | Treatment modality not meeting the inclusion criteria |

*Appendix 8.* Quality of Evidence (GRADE) / Summary of Findings Tables for (a) preclinical and (b) clinical trials.

| 1. **Summary of preclinical trials:** | | | | | | |
| --- | --- | --- | --- | --- | --- | --- |
| **Hyaluronic acid (HyA) compared to carrier for extraction socket** | | | | | | |
| **Patient or population: dogs, extraction sockets**  **Setting:**  **Intervention: HyA**  **Comparison: carrier** | | | | | | |
| Outcomes | **Anticipated absolute effects^*^** (95% CI) | | Relative effect (95% CI) | № of participants (studies) | Certainty of the evidence (GRADE) | Comments |
|  | **Bone formation with carrier** | **Bone formation with HyA intervention** |  |  |  |  |
| BV/TV after 3 months | - | MD **9.57 higher** (86.22 lower to 105.36 higher) | - | 24 (2 randomized experiments) | ⨁⨁◯◯ Low^a,b^ |  |
| ***The risk in the intervention group** (and its 95% confidence interval) is based on the assumed risk in the comparison group and the **relative effect** of the intervention (and its 95% CI).  **CI:** confidence interval; **MD:** mean difference | | | | | | |
| **GRADE Working Group grades of evidence** **High certainty:** we are very confident that the true effect lies close to that of the estimate of the effect. **Moderate certainty:** we are moderately confident in the effect estimate: the true effect is likely to be close to the estimate of the effect, but there is a possibility that it is substantially different. **Low certainty:** our confidence in the effect estimate is limited: the true effect may be substantially different from the estimate of the effect. **Very low certainty:** we have very little confidence in the effect estimate: the true effect is likely to be substantially different from the estimate of effect. | | | | | | |

#### Explanations

a. Both studies presented unclear risk of bias

b. Due to wide 95% CI

| 1. **Summary of clinical trials:** | | | | | | |
| --- | --- | --- | --- | --- | --- | --- |
| **Hyaluronic acid (HyA) compared to negative control or placebo/carrier for surgical LM3 removal** | | | | | | |
| **Patient or population: LM3 removal**  **Setting:**  **Intervention: HyA**  **Comparison: negative control or placebo/carrier** | | | | | | |
| Outcomes | **Anticipated absolute effects^*^** (95% CI) | | Relative effect (95% CI) | № of participants (studies) | Certainty of the evidence (GRADE) | Comments |
|  | **Risk with negative control or placebo/carrier** | **Risk with HyA Intervention** |  |  |  |  |
| Pain after 2-3 days | - | MD **0.52 SD lower** (0.34 higher to 1.38 lower) | - | 174 (4 RCTs) | ⨁⨁⨁◯ Moderate^a^ |  |
| Pain after 7 days | - | MD **0.32 SD lower** (0.12 lower to 0.51 lower) | - | 274 (5 RCTs) | ⨁⨁⨁◯ Moderate^b^ |  |
| Swelling after 2-3 days | - | MD **2.08 SD higher** (23.73 higher to 19.58 lower) | - | 68 (2 RCTs) | ⨁⨁◯◯ Low^b,c^ |  |
| Swelling after 7 days | - | MD **1.75 SD lower** (14.38 higher to 17.89 lower) | - | 68 (2 RCTs) | ⨁⨁◯◯ Low^b,c^ |  |
| Trismus after 2-3 days | - | MD **1.31 SD lower** (0.65 higher to 3.26 lower) | - | 128 (3 RCTs) | ⨁⨁⨁◯ Moderate^b^ |  |
| Trismus after 7 days | - | MD **1.08 SD lower** (0.97 higher to 3.12 lower) | - | 274 (5 RCTs) | ⨁⨁⨁◯ Moderate^b^ |  |
| ***The risk in the intervention group** (and its 95% confidence interval) is based on the assumed risk in the comparison group and the **relative effect** of the intervention (and its 95% CI).  **CI:** confidence interval; **MD:** mean difference | | | | | | |
| **GRADE Working Group grades of evidence** **High certainty:** we are very confident that the true effect lies close to that of the estimate of the effect. **Moderate certainty:** we are moderately confident in the effect estimate: the true effect is likely to be close to the estimate of the effect, but there is a possibility that it is substantially different. **Low certainty:** our confidence in the effect estimate is limited: the true effect may be substantially different from the estimate of the effect. **Very low certainty:** we have very little confidence in the effect estimate: the true effect is likely to be substantially different from the estimate of effect. | | | | | | |

#### Explanations

a. Two out of 4 studies presented some concerns

b. Half or most of the studies presented some concerns

c. Due to wide 95% CI
